# Curcumin and Its Analogue Induce Apoptosis in Leukemia Cells and Have Additive Effects with Bortezomib in Cellular and Xenograft Models

**DOI:** 10.1155/2015/968981

**Published:** 2015-05-17

**Authors:** L. I. Nagy, L. Z. Fehér, G. J. Szebeni, M. Gyuris, P. Sipos, R. Alföldi, B. Ózsvári, L. Hackler, A. Balázs, P. Batár, I. Kanizsai, L. G. Puskás

**Affiliations:** ^1^AVIDIN Ltd., Alsókikötő Sor 11, Szeged, Hungary; ^2^Department of Pharmaceutical Technology, University of Szeged, Eötvös u 6, Szeged 6720, Hungary; ^3^Department of Hematology, Institute of Internal Medicine, University of Debrecen, Nagyerdei Körút 98, Debrecen 4032, Hungary; ^4^Institute of Genetics, Biological Research Center of the Hungarian Academy of Sciences, Temesvári Körút 62, Szeged 6726, Hungary

## Abstract

Combination therapy of bortezomib with other chemotherapeutics is an emerging treatment strategy. Since both curcumin and bortezomib inhibit NF-*κ*B, we tested the effects of their combination on leukemia cells. To improve potency, a novel Mannich-type curcumin derivative, C-150, was synthesized. Curcumin and its analogue showed potent antiproliferative and apoptotic effects on the human leukemia cell line, HL60, with different potency but similar additive properties with bortezomib. Additive antiproliferative effects were correlated well with LPS-induced NF-*κ*B inhibition results. Gene expression data on cell cycle and apoptosis related genes, obtained by high-throughput QPCR, showed that curcumin and its analogue act through similar signaling pathways. In correlation with in vitro results similar additive effect could be obsereved in SCID mice inoculated systemically with HL60 cells. C-150 in a liposomal formulation given intravenously in combination with bortezomib was more efficient than either of the drugs alone. As our novel curcumin analogue exerted anticancer effects in leukemic cells at submicromolar concentration in vitro and at 3 mg/kg dose in vivo, which was potentiated by bortezomib, it holds a great promise as a future therapeutic agent in the treatment of leukemia alone or in combination.

## 1. Introduction

Curcumin, also known as diferuloyl methane, a natural component of the rhizome of* Curcuma longa* has emerged as one of the most powerful chemopreventive and anticancer agents. It has been reported to exert anti-inflammatory, antiangiogenic, and antiproliferative activity in various types of cancer [1–3]. It has also been shown that curcumin elevates intracellular ROS and induces loss of mitochondrial membrane potential and apoptosis in leukemia cells [4, 5]. In concert with these observations, the potent antileukemic action of curcumin and some of its derivatives have been reviewed recently [6]. This natural compound not only can be used as chemotherapeutics but also was suggested for chemoprevention [6].

A variety of molecular mechanisms have been proposed to mediate anticancer effects of curcumin, but its ability to inhibit the growth of various types of cancer cells at various stages of cancer progression is probably due to its potential to act on multiple targets [7–9]. Among others, it acts as a scavenger of free radicals [10], inhibits NF-*κ*B activation [11], and modulates histone deacetylase (HDAC) and histone acetyltransferase (HAT) enzyme activities [12, 13] and DNA methyltransferase 1 [10]. The main underlying action mechanisms of curcumin are probably based on the modulation of multiple important cellular signaling pathways including NF-*κ*B, TRAIL, PI3K/Akt, JAK/STAT, Notch-1, and JNK [5, 14].

Several preclinical and clinical studies suggest that curcumin may represent a novel strategy to treat cancer patients alone or in combination with already existing therapeutic regimens [2, 15]. However, the in vivo application of curcumin has been limited for its low potency and unsatisfactory bioavailability [16], which necessitates the application of new formulation solutions and the synthesis of novel curcumin analogues with improved pharmacological properties, while retaining a similar safety profile.

Different synthetic concepts have been therefore developed to expand the molecular diversity, from the side-chain and diketone transformations to alkyl and alkenyl functionalizations on C-4 in the central position or with different substituents at the 4 positions of the phenyl group of curcumin [17–20]. Here, we report the application of a novel Mannich-type curcumin derivative, C-150, possessing metahydroxyphenyl side-chains and 3-phenyl-3-acrylamido branched central motif. The new analogue is one of the most potent members of the synthesized analogues [21].

Previous reports demonstrated that bortezomib (Velcade, formerly known as PS-341), a potent and selective proteasome inhibitor approved by the FDA for the treatment of patients with multiple myeloma, is able to block chemotherapy-induced NF-*κ*B activation and augment the apoptotic response to chemotherapeutic agents [22].

NF-*κ*B signaling plays a critical role in cancer development and progression; constitutive activation of NF-*κ*B pathway has been reported in different types of cancer, including multiple myeloma and acute myeloid leukemias [23, 24]. After stimulation and activation of the canonical NF-*κ*B pathway in noncancer cells, I*κ*B is phosphorylated by IKK then degradated by the 26S proteasome allowing the nuclear transport of NF-*κ*B. Since I*κ*B is a substrate of the proteasome, the initial rationale for use of bortezomib in multiple myeloma was inhibition of NF-*κ*B activity and inhibition of cell proliferation and survival [25, 26].

Beside the canonical pathway, several studies show a critical role for the noncanonical NF-*κ*B pathway in cancer pathogenesis as well. Hideshima et al. found that bortezomib could inhibit the noncanonical pathway via inhibition of conversion of p100 to p52 and its nuclear translocation in multiple myeloma cells [27].

It has been shown that bortezomib possesses in vitro and in vivo activity against a variety of malignancies, including acute and chronic lymphocytic leukemia, prostate cancer, pancreatic cancer, and colon cancer [22]. Combination therapy of bortezomib with other chemotherapeutical agents is an emerging treatment strategy not only in myeloma but also in leukemia [28]. Since both curcumin and bortezomib inhibit NF-*κ*B, we hypothesized their additive anticancer effects on leukemia cells.

Several studies reported enhanced cytotoxicity of bortezomib in multiple myeloma cells when combined with curcumin or its analogues both in vitro and in vivo [28–31]. In multiple myeloma cells, the combined treatment inactivated NF-*κ*B while it activated JNK signaling [31], but no information has been available on leukemia cells.

In this report, we studied curcumin and a novel Mannich-type curcumin analogue C-150 alone and in combination with bortezomib. Effects of cotreatment were studied on cytotoxicity, NF-*κ*B inhibition, gene expression, and in an in vivo mouse model.

## 2. Materials and Methods

### 2.1. Cell Culturing

Human leukemia cells (HL60) were obtained from ATCC, USA. Cells were cultured at 37°C under 5% CO_2_ and 100% humidity in LeviTubes designed for use in the bench top bioreactor-incubator hybrid BioLevitator (Hamilton, Hungary). During cultivation, cells were proliferated in suspension with rotation without the use of microcarriers. Rotation period was 2 seconds with 1 second pause at 65 rpm. LeviTubes were filled with 40 mL RPMI cell culture medium containing 10% fetal bovine serum, 1x GlutaMAX, and penicillin-streptomycin antibiotics (Life Technologies, Hungary). Culture medium was changed after 48 hours by removing half of the volume of the culture medium and replacing it with fresh medium.

### 2.2. Cell Viability Measurements

Viability of HL60 human leukemia cells was determined by the colorimetric MTS assay (CellTiter 96 AQueous Assay, Promega, Madison, WI) and by the CellTiter-Glo luminescent cell viability assay (Promega, Madison, WI). The cells were seeded into 96-well plates at 7000 cells/well density and cultured overnight before treatment. Effects of curcumin (5, 10, 25, and 50 *μ*M), C-150 (50, 100, and 500 nM and 1 *μ*M), bortezomib (0.78, 3.125, 12.5, and 50 nM), and their combinations were recorded 48 hours after treatment. MTS reagent (3-(4,5-dimethylthiazol-2-yl)-5-(3-carboxymethoxy-phenyl)-2-(4-sulfophenyl)-2H-tetrazo-lium) or CellTiter-Glo reagent was applied to drug treated and control (0.2% DMSO) cells according to the manufacturer's protocol. Absorbance (at 490 nm) and luminescence were recorded on a multimode microplate reader (Victor, Perkin Elmer).

Viability was calculated with relation to untreated control cells from three parallel measurements for all conditions. For statistical significance, two-tailed Student's *t*-test was used.

### 2.3. Measurements of NF-*κ*B Inhibition

Mouse B16 melanoma cell line was obtained from ATCC, USA. Cells were cultured in RPMI medium (Life Technologies, Hungary) supplemented with 10% FCS (Life Technologies, Hungary). NF-*κ*B reporter cell lines were created by transfection with the pNF-*κ*B-Luc/neo reporter construct with the Lipofectamine 2000 reagent (Invitrogen) and stable cell lines were selected by G418 (Sigma) treatment.

B16/NF-*κ*B-Luc cells (6 × 10^4^ cells/well) were grown on luminoplates (Corning Life Sciences, Kentucky, USA) overnight under standard cell culturing conditions. Before treatment with curcumin (5, 10, 25, and 50 *μ*M), C-150 (50, 100, and 500 nM and 1 *μ*M), bortezomib (0.78, 3.125, 12.5, and 50 nM) and their combinations, the NF-*κ*B-Luc reporter gene was induced by adding LPS (500 ng/mL) into the cell culture media (500 ng/mL). After 6 hours of incubation with curcumin, C-150, bortezomib, or their combinations, the medium was removed; cells were washed and lysed for 10 min at room temperature in cell culture lysis reagent (20 *μ*L/well; Promega). Substrate was added (20 *μ*L/well; Promega), and luciferase activity was measured in a microplate reader (Victor, Perkin Elmer). NF-*κ*B inhibition was calculated with relation to untreated control cells from three parallel measurements for all conditions.

### 2.4. FACS Analysis

HL60 cells (10^5^) were plated in 24-well tissue culture plates (Corning Life Sciences). Cells were treated with curcumin (25 *μ*M), C-150 (300 and 600 nM), bortezomib (20 nM), and their combinations in triplicates. After 48 hours, cells were collected and resuspended in annexin V binding buffer (0.01 M HEPES, 0.14 M NaCl, and 2.5 mM CaCl_2_). Annexin V-Alexa 488 (Life Technologies, 2.5 : 100) and propidium iodide (PI) (Sigma-Aldrich, 10 *μ*g/mL) were added to the cells and placed for 15 min in dark at room temperature. After washing, the cells were analyzed on the FACSCalibur cytofluorimeter (Becton Dickinson). Percentage of FL1 (annexin V-Alexa 488) positive and FL3 (propidium iodide) negative early apoptotic cells and FL1 (annexin V-Alexa 488) positive and FL3 (propidium iodide) positive late apoptotic cells was determined. For statistical significance, two-tailed Student's *t*-test was used.

### 2.5. Gene Expression Analysis by High-Throughput QPCR

HL60 leukemia cells (10^6^) were plated on 10 cm^2^ tissue culture plates (Corning Life Sciences). Cells were treated with curcumin, C-150, bortezomib, and their combinations. 48 h after treatment, cells were collected and centrifuged (3000 rpm, 5 min) and total RNA was purified from treated and control (0.2% DMSO) cells using AccuPrep Viral RNA Extraction kit (Bioneer Corp., Korea) with a modified protocol as described earlier [32]. Briefly, cells were lysed with RA1 lysis buffer (Macherey-Nagel, Düren, Germany) and applied to the Viral RNA Extraction binding tube and then washed and eluted with the protocol recommended by the manufacturer. The quantity of total RNA was measured by NanoDrop 1000 spectrophotometer. 6 *μ*g total RNA was converted into cDNA with the High-Capacity cDNA Archive Kit (Applied Biosystems, Foster City, CA) in a total volume of 60 *μ*L.

Identical reaction volumes were prepared by the Agilent Bravo Liquid handling Platform (Agilent Technologies) in a 16 × 96-well design according to Agilent and Roche's recommendations. Each 2 *μ*L reaction mixture contained 6 ng cDNA, 10 pmole gene specific primers, and 1 *μ*L 2x LightCycler1536 Probes Master (Roche). The following amplification protocol was used: 95°C for 1 minute (activation), 60 cycles of 95°C for 10 seconds, and 60°C for 10 seconds, followed by 40°C for 10 second final cooling. Amplification was performed on the LightCycler 1536 System (Roche Applied Science) using the real-time ready human cell cycle regulation panel and the real-time ready human apoptosis panel (Roche Applied Science). Each panel consists of 84 cell cycles and apoptosis related assays along with seven human reference genes (ACTB, *β*2M, GAPDH, HPRT1, RPL13A, 18S, and YWHAZ).

Data was collected and processed using the LightCycler 1536 SW 1.0 software. Curves were analyzed by using dynamic tube and slope correction methods. Relative expression of the analyzed genes was normalized to the mean value of the reference genes.

### 2.6. Liposome Formulation of C-150

Liquid phase C-150 containing liposomes was produced by dissolving lipid powder of CHOL/PC/DSPE-mPEG (62/28/0.6 mol%) and C-150 (9.2 mol%) in ethanol. The solution was transferred to a rotary evaporator and dried overnight in vacuum. The lipid film was hydrated and redispersed in PBS solution to a final C-150 concentration of 0.5 mM for 25 min over the phase transition temperature of the organic components. The dispersion was subjected to size extrusion (0.45 *μ*m pore size) and finally filtered through sterile filters (0.22 *μ*m pore size). After preparation, droplet size and polydispersity index of the liposomes were determined. The droplet size, SSA, and PDI of liposomes were measured by laser diffractometry (*n* = 5) by the wet method. The d0.1, d0.5 as median and d0.9 droplet size values, SSA, and PDI values were 75 ± 19 nm, 114 ± 20 nm, 178 ± 24 nm, 54.5 m^2^/g, and 0.901 ± 0.20, respectively.

### 2.7. In Vivo Effects of C-150 and Bortezomib in a Xenograft Model

8-week-old male NOD CB17-Prkdc^scid^/NcrCrl mice (Charles River, Innovo Kft., Hungary) were used. The animals were kept in individual ventilated cages to avoid any infection. All mice were fed a commercial sterile diet and water ad libitum and were housed in an animal facility under a 12 h light/dark cycle at constant temperature and humidity.

For the induction of rodent model of human leukemia 10^6^ HL60, human leukemia cells were injected in 100 *μ*L volume intravenously. 3 days after cancer cell inoculation mice were randomized and treated 5 times a week for 2 weeks.

Four different groups, each containing 8 mice, were studied: PBS control, bortezomib (0.15 mg/kg), C-150 (3 mg/kg), and C-150 (3 mg/kg) + bortezomib (0.15 mg/kg). All animals were treated moribund and were euthanized at the observation of the first sign of torment. The study was performed according to the Institutional and National Animal Experimentation and Ethics Guidelines in possession of an ethical clearance (XXIX./3610/2012).

### 2.8. Statistical Analysis

All data presented are means ± standard deviation (SD) as indicated in the text. The statistical comparisons were performed by two-tailed Student's *t*-test using Microsoft Excel. In all statistical comparisons, a probability (*P*) value of less than 0.05 was considered significant.

## 3. Results and Discussion

### 3.1. In Vitro Effect of Curcumin and Its Analogue Alone and in Combination with Bortezomib

We have utilised two common viability assays to assess the cytotoxic effects of curcumin and the novel analogue C-150 on HL60 cells. Combinations of curcumin or C-150 with bortezomib were also investigated. The colorimetric MTS assay and the luminescence based CellTiter-Glo (CTG) assay yielded comparable results; however, additive effects could be registered at different concentrations (Tables 1 and 2). We found that the CellTiter-Glo assay resulted in additive effects at lower concentrations both in case of curcumin and C-150, which can be explained by the difference in sensitivity of the two assays. While MTS assay measures the activity of NAD(P)H-dependent cellular oxidoreductase enzymes of the mitochondria, the CellTiter-Glo assay detects cellular ATP content.

We found that curcumin had a dose-dependent cytotoxic effect on HL60 cells (MTS IC_50_ = 8.21 *μ*M CTG: IC_50_ = 16.77 *μ*M). C-150 also exhibited a dose-dependent effect on HL60 cells but with 27–60-fold higher potency than curcumin (MTS IC_50_ = 0.30 *μ*M, CTG IC_50_ = 0.27 *μ*M). In the combination experiments, bortezomib was used at nanomolar concentrations and the determined IC_50_ value of bortezomib alone was 6.56 and 6.90 nM in the MTS and the CTG assays, respectively.

At the highest applied concentrations, both curcumin and bortezomib alone produced robust cell death, which could not be further enhanced by the combined treatment. At the lowest applied concentration, both curcumin and bortezomib showed no cytotoxic effect neither alone nor in combination. Interestingly, only the CTG assay detected appreciable and statistically significant additive effects. Viability dramatically decreased after combination of 10 *μ*M curcumin and 3.125 nM bortezomib, by 47 and 39% compared to curcumin or bortezomib alone. Similarly, combination of 5 *μ*M curcumin and 3.125 nM bortezomib also decreased viability compared to either of the compounds alone (see Table 2).

Combination of C-150 and bortezomib also showed additive effects. Both methods detected a statistically significant decrease in viability following the combination of 0.1 *μ*M of C-150 and 3.125 nM of bortezomib. Again, more dramatic changes were detected by the CTG assay than MTS.

In conclusion, cytotoxic effects of curcumin and its analogue were detected by two cell viability measurements. Furthermore, we showed that both curcumin and C-150 additively potentiated the effect of bortezomib.

### 3.2. In Vitro Effects on the NF-*κ*B Pathway

In the same concentration ranges as in the viability tests, we determined the effect of curcumin, C-150, and bortezomib on NF-*κ*B induction inhibition. All three compounds decreased the activation of NF-*κ*B markedly in a dose-dependent manner. IC_50_ values were 9.6 *μ*M for curcumin, 0.8 *μ*M for C-150, and 2.0 nM for bortezomib. Significant additive effects could not be recorded for curcumin and bortezomib while, in case of C-150, a modest additive effect was found in some concentration combinations (Table 3).

Several studies show a critical role for the NF-*κ*B pathway in multiple myeloma and leukemia pathogenesis as well [23, 24]. Hideshima et al. found that bortezomib triggers the canonical pathway of NF-*κ*B and downregulates I*κ*B*α* in primary cells from patients with MM. However, they also found that bortezomib could inhibit the noncanonical pathway via inhibition of conversion of p100 to p52 and its nuclear translocation in MM.1S cells [27]. It was also shown that the main underlying mechanisms of action of curcumin were probably based on the modulation of multiple important cellular signaling pathways including NF-*κ*B [5, 14]. Here, we demonstrated that both types of anticancer agents inhibited NF-*κ*B induction and curcumin and C-150, in concordance with their additive cytotoxic potential confirmed by our in vitro studies, which potentiated the effects of bortezomib.

### 3.3. Apoptosis Detection

Using FACS analysis and annexin V and PI staining, the percentage of cells in early or late apoptosis was determined.

Two doses of curcumin (15 and 25 *μ*M) and C-150 (0.3 and 0.6 *μ*M) were applied. Bortezomib was applied at 20 nM concentration. Both curcumin and C-150 at their higher concentration induced 100% and 70% programmed cell death, respectively. No additive effect could be observed when bortezomib was coadministered (Table 4).

**Figure 1:**
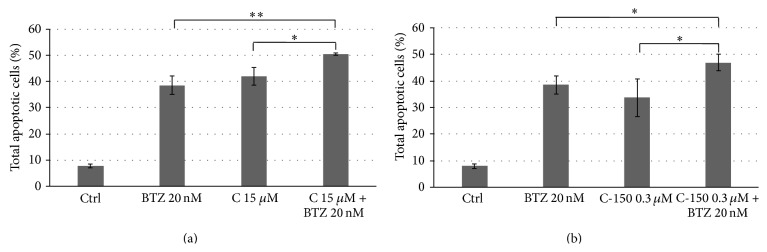
Effect of curcumin (C) or C-150 treatments alone or in combination with bortezomib (BTZ) on cell viability. C-150 (0.3 *μ*M) could potentiate the cytotoxic effect of 20 nM bortezomib at 50-fold lower concentration than curcumin (15 *μ*M). Data represent total apoptotic cells (^*^ *P* ≤ 0.05; ^**^ *P* ≤ 0.01, two-tailed Student's *t*-test).

At the lower curcumin concentration (15 *μ*M), we could detect a statistically significant additive effect between curcumin and bortezomib (Figure 1(a)). C-150 at 0.3 *μ*M (50 times lower concentration) possessed a similar apoptotic effect compared to curcumin at 15 *μ*M (Figure 1(b)). Coadministration of bortezomib also showed a significant additive effect (Figure 1(b)) with 0.3 *μ*M C-150.

From these results, we can conclude that the novel curcumin analogue induced a similar level of apoptosis at 50-fold lower concentration than curcumin, while retaining the ability to potentiate the effect of bortezomib. In addition, we found that both curcumin and C-150 induced apoptosis and not necrosis, since the only propidium iodide positive population did not change (data not shown).

### 3.4. Analysis of Apoptosis and Cell Cycle Related Gene Expression Affected by Combination Treatments

**Figure 2:**
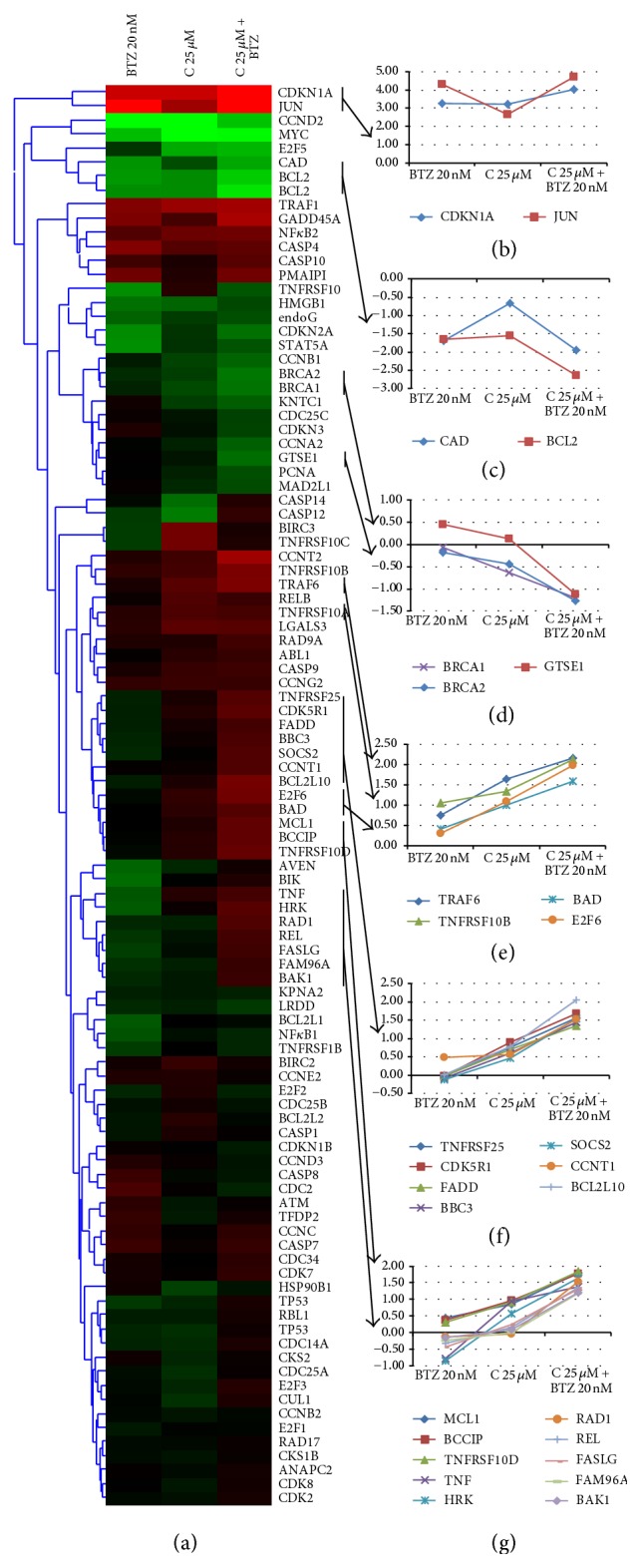
Gene expression analysis of curcumin (C) and bortezomib (BTZ) treatment. Curcumin influenced the expression of a wide range of cell cycle and apoptosis related genes.

We have analysed the effects of curcumin on the expression of apoptosis and cell cycle related genes using high-throughput QPCR.  Figure 2 depicts the hierarchical cluster analysis of the obtained gene expression patterns. Previous studies highlighted that treatment with curcumin and bortezomib activated JNK signaling in multiple myeloma cells [33]. In agreement with that, we observed increased expression of JUN (jun protooncogene), a target of JNK (MAPK8, mitogen-activated protein kinase 8), following both curcumin and bortezomib treatment, and this overexpression was enhanced by their combined use. A negatively regulated target of JNK signaling BCL2 (B-cell CLL/lymphoma 2) was also affected; it was downregulated by curcumin and bortezomib and by their combination. Here, a significant additive effect was also observed. (b), (c), (d), (e), (f), and (g) panels of Figure 2 depict representative genes of the identified clusters. Panel (b) contains genes that were activated by both curcumin and bortezomib with additive effects following the combined treatment. Panels (c) and (d) contain genes that were markedly downregulated: by all treatments (Panel (c)) or by only the combined treatment (Panel (d)). Panels (e), (f), and (g) plot those genes that were upregulated following combined treatment, suggesting a role in the observed additive effects between curcumin and bortezomib.

We can conclude that both apoptosis and cell cycle related genes were differentially expressed by curcumin and by its combination with bortezomib.

**Figure 3:**
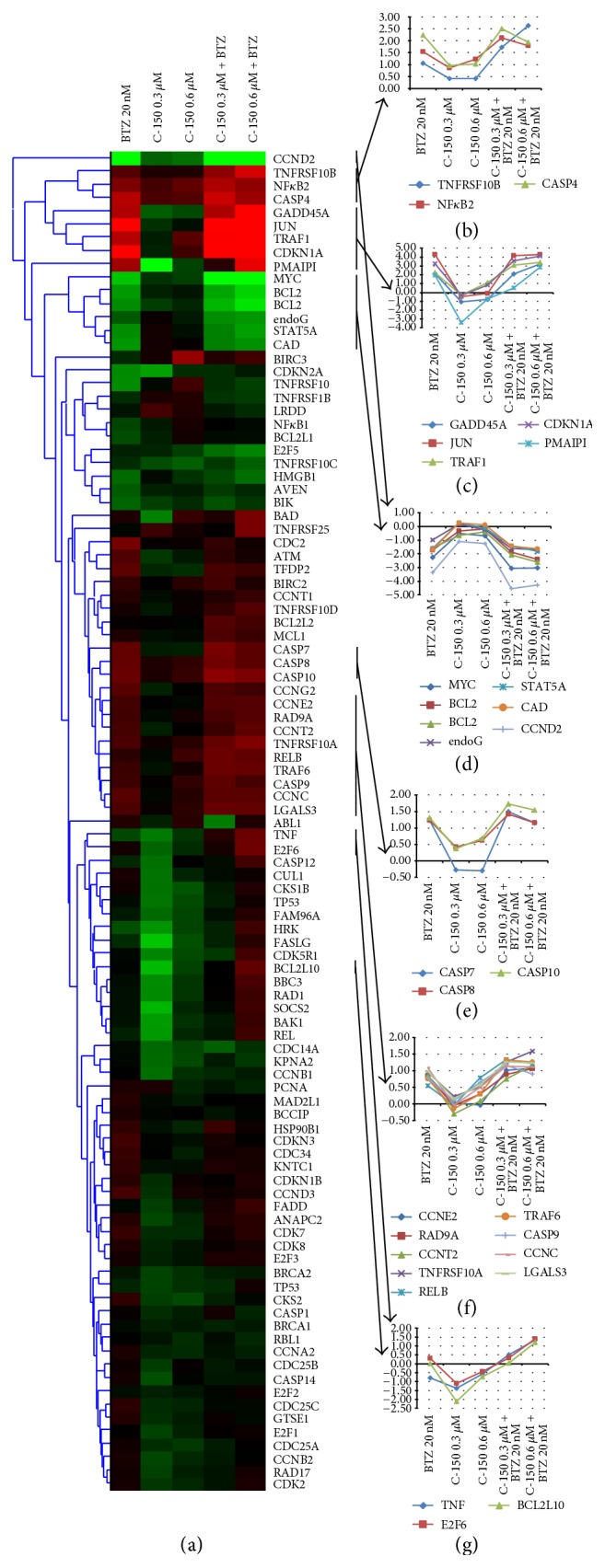
Gene expression analysis of curcumin analogue C-150 and bortezomib treatment. C-150 influenced the expression of a wide range of cell cycle and apoptosis related genes at 40–80-fold lower concentration compared to curcumin.

We have also investigated the effects of the novel analogue C-150 alone and in combination on genes related to apoptosis and cell cycle.  Figure 3 summarizes the results of hierarchical cluster analysis and highlights representative genes of the identified gene expression patterns.

In conclusion, C-150 possessed dose-dependent effects on certain apoptosis and cell cycle related genes which could be enhanced by coadministration with bortezomib. In comparison with cotreatment of bortezomib and curcumin or C-150, similar gene expression changes were detected, which suggests a similar mechanism of action.

### 3.5. Common Genes Influenced by Both Curcumin and C-150

**Figure 4:**
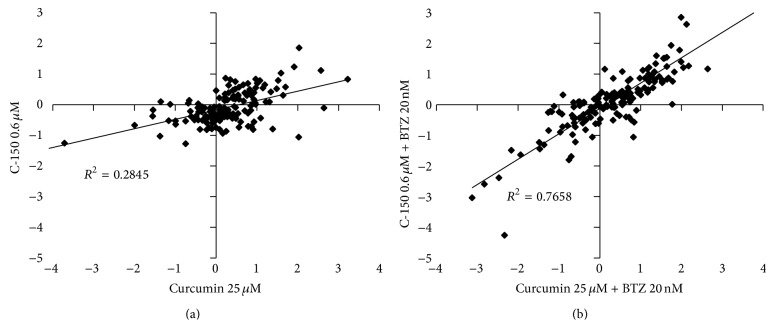
Correlation of gene expression changes following curcumin and C-150 treatment (a). Correlation of gene expression values of the combined treatments of curcumin + bortezomib and C-150 (0.6 *μ*M) + bortezomib (b). The natural compound curcumin and its synthetic analogue C-150 affected nearly the same genes in a similar fashion, but curcumin analogue exerted its effect at 40-fold lower concentration.

When gene expression values from curcumin and C-150 treated cells were plotted against each other and their correlation was investigated, we concluded that genes most affected by treatment behaved in a similar fashion suggesting that the related compounds act with similar mechanism of action (Figure 4). Several cyclins were unaffected by treatment but two previously described genes CCND1 (cyclin D1) and CCND2 (cyclin D2) [34] were both downregulated by curcumin and C-150. BIRC3 (BIRC3 baculoviral IAP repeat containing 3) and CASP4 (caspase 4, apoptosis-related cysteine peptidase) expression was also activated by both compounds agreeing with previous reports [35, 36].

While fewer genes responded to C-150 treatment alone compared to curcumin alone (with at least 2-fold induction or repression), combined treatment of C-150 and bortezomib affected the same genes with a similar profile as curcumin and bortezomib together (Figure 4(b)). Similar to viability measurements with MTS or CTG and FACS analysis, C-150 induced comparable results to curcumin at more than 40 times less concentration (25 *μ*M versus 0.6 *μ*M).

### 3.6. Genes Influenced Differentially by Curcumin and C-150

It seems that, among the tested members of the tumor necrosis factor receptor superfamily TNFRSF10A, B and C were differentially affected by curcumin and C-150. All three genes were upregulated by curcumin, while C-150 did not alter their expression. GADD45A (growth arrest and DNA-damage-inducible, alpha) was also differentially affected by curcumin and its analog. This stress response gene also mediates the activation of the JNK pathway [37], which also showed difference in the expression of JUN that was upregulated by curcumin and was not affected by C-150. These differences may rise from differences of the two compounds in target affinity or more likely effectiveness of the applied concentrations. Further studies are needed to validate these findings.

### 3.7. In Vivo Effects of Liposome-Formulated C-150 and Bortezomib Alone and in Combination

The effective therapy of acute myelocytic leukemia still remains problematic. Single treatment of leukemia and similar hematological cancer types has shown modest improvement. Despite chemotherapy, nearly half of the patients with myelocytic leukemias will fall prey to the disease because of the maturation of multiresistant subclones of leukemia cells [38, 39]. Numerous studies aimed to find effective compounds that are coadministered with chemotherapeutic agents which can increase efficacy of the treatment and lower its side effects. Several studies concluded that the sensitivity of some cancer cells can be increased by inhibiting several intracellular pathways in parallel which finally result in apoptosis. From this evidence, it can make sense to use the conventional chemotherapeutic agents in combination with other compounds that can sensitize the target cells.

We found that curcumin and its synthetic analogue C-150 can improve the effect of bortezomib in vitro. From the evidence published by Bai and Zhang and from our findings, curcumin and its analogue C-150 can modulate and inactivate the NF-*κ*B pathway which is necessary to cell survival and activates the JNK pathway an activator of apoptosis [33]. In order to prove our hypothesis that C-150 could increase the effect of bortezomib against promyelocytic leukemia in vivo also, a study using a rodent model system was performed in SCID mice. In our in vivo study, 8 mice were used in 4 groups.

**Figure 5:**
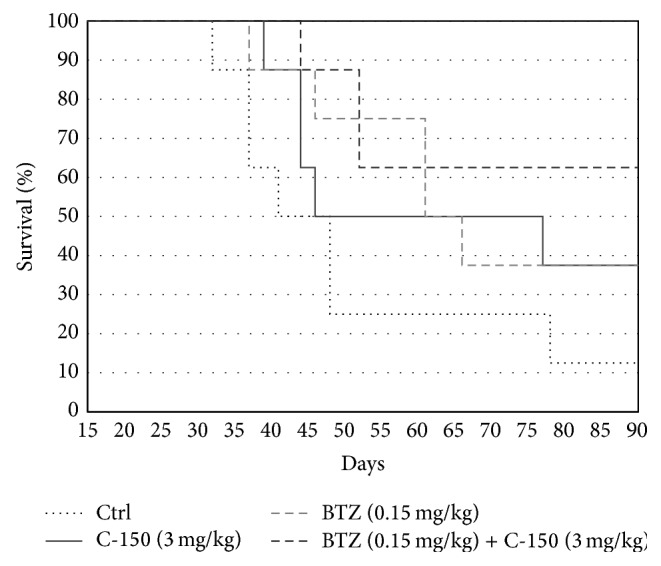
Survival of HL-60 xenograft mice treated with C-150 (3 mg/kg), bortezomib (0.15 mg/kg), or their combination. The curcumin analogue C-150 and bortezomib alone exhibited antitumor activity in HL-60 xenograft SCID mice, whereas combined treatment (bortezomib + C-150) developed a greater antitumor activity compared to single treatments.

We found that 1/8 mice survived in the control group, while both bortezomib and C-150 treatment alone was effective; 3/8 survived after 90 days in both groups. When C-150 and bortezomib were administered in combination, 5/8 mice survived. In addition, we found that this survival ratio did not change until 120 days, when the experiment was terminated (Figure 5).

Consistent with the results obtained in vitro, bortezomib or liposome formulated C-150 alone exhibited antitumor activity in HL-60 xenograft SCID mice, whereas combined bortezomib and C-150 treatment resulted in greater antitumor activity than single treatments. These results suggest that the combined use of bortezomib and C-150 could be developed into an effective option in the treatment of AML.

## 4. Conclusion

The multitarget action of curcumin and its analog, C-150, showed additive cytotoxic effects with bortezomib. Curcumin and its analogue induced expression changes in apoptotic and cell cycle related genes as determined by high-throughput QPCR. Most of the affected genes showed similar changes suggesting that the related compounds act through similar signaling pathways. Differences in tumor necrosis factor receptor superfamily and GADD45a gene activity profiles induced by curcumin and C-150 could be explained by differences in their target affinity or more likely, effectiveness of the applied concentrations. The enhanced proliferation inhibition capability of curcumin and C-150 analogue with bortezomib was confirmed by in vitro as well as in vivo studies. Pronounced positive effects of the liposomal formulated curcumin analogue on survival of HL60 xenograft mice would render this molecule a potent clinical candidate against leukemia alone or in combination with other antineoplastic agents.

## Supplementary Material

"Supplementary Table: Normalized gene expression values (∆∆Ct) of all tested conditions. Curcumin and its analogue (C-150) induced expression changes in apoptotic and cell-cycle related genes as determined by high-throughput QPCR. Most of the affected genes showed similar changes suggesting that the related compounds act through similar signaling pathways. C-150 possessed dose dependent effects on certain apoptosis and cell cycle related genes in 40-fold lower concentration than curcumin which could be enhanced by coadministration with bortezomib. Red cells: at least 2 fold induction, green cells: at least 2 fold repression, nd: not detected transcript.

## Figures and Tables

**Table 1:** Cell viability measurements (MTS) of curcumin (C), C-150, and bortezomib (BTZ) alone and combined. Curcumin and its analogue C-150 and bortezomib significantly decreased the cell viability of HL-60 human leukemia cells in a dose-dependent manner. C-150 exhibited dose-dependent effect on HL60 cells in submicromolar concentrations and was effective at 27 times lower concentration than curcumin. Both curcumin and its analogue exhibited additive effects with bortezomib (bold data with significant values) (*P*_1_: bortezomib versus combination; *P*_2_: curcumin or C-150 versus combination; two-tailed Student's *t*-test).

	—	BTZ (50 nM)	BTZ (12.5 nM)	BTZ (3.125 nM)	BTZ (0.78 nM)
—		0.35	0.45	1.26	1.34
C (50 *µ*M)	0.34	0.33	0.35	0.39	0.37
C (25 *µ*M)	0.26	0.23	0.31	0.28	0.27
C (10 *µ*M)	0.48	**0.28** (**P**_1_ < 0.05, **P**_2_ < 0.01)	**0.31** (**P**_1_ < 0.05, **P**_2_ < 0.01)	0.42	0.60
C (5 *µ*M)	1.10	0.33	0.43	1.21	1.26
C-150 (1 *µ*M)	0.25	0.21	**0.19** (**P**_1_ < 0.01, **P**_2_ < 0.05)	0.23	0.25
C-150 (0.5 *µ*M)	0.40	**0.26** (**P**_1_, **P**_2_ < 0.01)	0.27	**0.27** (**P**_1_ < 0.01, **P**_2_ < 0.05)	0.33
C-150 (0.1 *µ*M)	0.94	0.25	0.41	0.83	1.02
C-150 (0.05 *µ*M)	1.16	0.32	0.39	1.13	1.22

**Table 2:** Cell viability measurements of curcumin (C), C-150, and bortezomib (BTZ) alone and in combination with CellTiter-Glo assay. Curcumin and its analogue C-150 significantly potentiated the effect of bortezomib (*P*_1_: bortezomib versus combination; *P*_2_: curcumin or C-150 versus combination; two-tailed Student's *t*-test).

	—	BTZ (50 nM)	BTZ (12.5 nM)	BTZ (3.125 nM)	BTZ (0.78 nM)
—		0.11	0.16	0.91	0.94
C (50 *µ*M)	0.12	0.11	0.12	0.12	0.11
C (25 *µ*M)	0.17	0.15	0.13	**0.13** (**P**_1_ < 0.01, **P**_2_ < 0.05)	**0.13** (**P**_1_ < 0.01, **P**_2_ < 0.05)
C (10 *µ*M)	0.99	0.11	0.15	0.52 (**P**_1_, **P**_2_ < 0.01)	0.87
C (5 *µ*M)	1.08	0.11	0.17	0.79	1.05
C-150 (1 *µ*M)	0.09	0.10	**0.10** (**P**_1_ < 0.01, **P**_2_ < 0.05)	0.08	0.08
C-150 (0.5 *µ*M)	0.16	0.10	0.10	0.12	0.16
C-150 (0.1 *µ*M)	0.99	**0.08** (**P**_1_ < 0.05, **P**_2_ < 0.01**)**	0.12 (**P**_1_ < 0.05, **P**_2_ < 0.01)	**0.58** **(** **P**_1_, **P**_2_ < 0.01**)**	0.90
C-150 (0.05 *µ*M)	0.93	0.10	0.15	**0.69** (**P**_1_ < 0.01, **P**_2_ < 0.05)	1.02

**Table 3:** In vitro effect of curcumin and C-150 on NF-*κ*B signaling. Curcumin and its analogue C-150 alone or in coadministration with bortezomib decreased the activation of NF-*κ*B pathway in a dose-dependent manner. Modest significant additive effect was recorded after coadministration of C-150 with bortezomib (*P*_1_: bortezomib versus combination; *P*_2_: curcumin or C-150 versus combination; two-tailed Student's *t*-test).

	—	BTZ (50 nM)	BTZ (12.5 nM)	BTZ (3.125 nM)	BTZ (0.78 nM)
—		0.36	0.44	0.54	0.94
C (50 *µ*M)	0.19	0.20	0.22	0.28	0.28
C (25 *µ*M)	0.20	0.24	0.31	0.44	0.34
C (10 *µ*M)	0.43	0.32	0.25	0.47	0.54
C (5 *µ*M)	1.05	0.54	0.61	0.64	0.94
C-150 (1 *µ*M)	0.58	0.37	0.41	**0.44** (**P**_1_ < 0.01, **P**_2_ < 0.05)	0.65
C-150 (0.5 *µ*M)	0.82	0.36	0.50	0.56	0.72
C-150 (0.1 *µ*M)	1.17	**0.32** (**P**_1_ < 0.05, **P**_2_ < 0.01)	0.54	0.58	**0.79** (**P**_1_ < 0.05, **P**_2_ < 0.01)
C-150 (0.05 *µ*M)	0.99	0.38	0.54	**0.49** (**P**_1_, **P**_2_ < 0.01)	0.95

**Table 4:** Summary of FACS analysis. Percentage of cells in early and late stages of apoptosis and total apoptotic cells as determined by annexin V and PI staining. The curcumin analogue C-150 induced a similar rate of apoptosis at 50-fold lower concentration than curcumin. Furthermore, it retained the ability to potentiate the effect of bortezomib.

	Early apoptotic cells Annexin V+/PI−		Late apoptotic cells Annexin V+/PI+		Total apoptotic cells	
	%	+/−	%	+/−	%	+/−
Control	4.5	0.5	3.4	0.6	7.9	1.1
BTZ 20 nM	21.4	0.4	16.5	4.2	37.9	4.6
C 15 *µ*M	18.6	0.8	21.9	4.2	40.5	5.0
C 15 *µ*M + BTZ 20 nM	21.3	1.3	29.1	1.6	50.4	2.9
C 25 *µ*M	45.6	5.0	54.1	4.9	99.6	9.9
C 25 *µ*M + BTZ 20 nM	53.3	8.0	46.5	7.9	99.7	15.9
C-150 0.3 *µ*M	23.8	4.1	7.0	2.7	30.8	6.9
C-150 0.3 *µ*M + BTZ 20 nM	25.4	0.1	21.4	4.4	46.9	4.5
C-150 0.6 *µ*M	24.5	1.0	45.9	3.9	70.4	4.9
C-150 0.6 *µ*M + BTZ 20 nM	24.2	2.0	45.7	3.2	69.8	5.3

## References

[1] Fresco P., Borges F., Marques M. P. M., Diniz C. (2010). The anticancer properties of dietary polyphenols and its relation with apoptosis. *Current Pharmaceutical Design*.

[2] Aggarwal B. B., Kumar A., Bharti A. C. (2003). Anticancer potential of curcumin: preclinical and clinical studies. *Anticancer Research*.

[3] Arbiser J. L., Klauber N., Rohan R. (1998). Curcumin is an in vivo inhibitor of angiogenesis. *Molecular Medicine*.

[4] Gopal P. K., Paul M., Paul S. (2014). Curcumin induces caspase mediated apoptosis in JURKAT cells by disrupting the redox balance. *Asian Pacific Journal of Cancer Prevention*.

[5] Guo Y., Shan Q., Gong Y. (2014). Curcumin induces apoptosis via simultaneously targeting AKT/mTOR and RAF/MEK/ERK survival signaling pathways in human leukemia THP-1 cells. *Die Pharmazie*.

[6] Kelkel M., Jacob C., Dicato M., Diederich M. (2010). Potential of the dietary antioxidants resveratrol and curcumin in prevention and treatment of hematologic malignancies. *Molecules*.

[7] Aggarwal B. B., Sung B. (2009). Pharmacological basis for the role of curcumin in chronic diseases: an age-old spice with modern targets. *Trends in Pharmacological Sciences*.

[8] Kunnumakkara A. B., Anand P., Aggarwal B. B. (2008). Curcumin inhibits proliferation, invasion, angiogenesis and metastasis of different cancers through interaction with multiple cell signaling proteins. *Cancer Letters*.

[9] Yu J., Peng Y., Wu L.-C. (2013). Curcumin down-regulates DNA methyltransferase 1 and plays an anti-leukemic role in acute myeloid leukemia. *PLoS ONE*.

[10] Menon V. P., Sudheer A. R. (2007). Antioxidant and anti-inflammatory properties of curcumin. *Advances in Experimental Medicine and Biology*.

[11] Singh S., Khar A. (2006). Biological effects of curcumin and its role in cancer chemoprevention and therapy. *Anti-Cancer Agents in Medicinal Chemistry*.

[12] Balasubramanyam K., Varier R. A., Altaf M. (2004). Curcumin, a novel p300/CREB-binding protein-specific inhibitor of acetyltransferase, represses the acetylation of histone/nonhistone proteins and histone acetyltransferase-dependent chromatin transcription. *The Journal of Biological Chemistry*.

[13] Li X.-G., Chen Y., Wu Q., Liu H.-L. (2005). Effects of curcumin on the acetylation of histone H3, P53 and the proliferation of NB4 cells. *Zhonghua Xue Ye Xue Za Zhi*.

[14] Chen J., Wang F. L., Chen W. D. (2014). Modulation of apoptosis-related cell signalling pathways by curcumin as a strategy to inhibit tumor progression. *Molecular Biology Reports*.

[15] Hatcher H., Planalp R., Cho J., Torti F. M., Torti S. V. (2008). Curcumin: from ancient medicine to current clinical trials. *Cellular and Molecular Life Sciences*.

[16] Anand P., Kunnumakkara A. B., Newman R. A., Aggarwal B. B. (2007). Bioavailability of curcumin: problems and promises. *Molecular Pharmaceutics*.

[17] Wu L. X., Wu Y., Chen R. J. (2014). Curcumin derivative C817 inhibits proliferation of imatinib-resistant chronic myeloid leukemia cells with wild-type or mutant Bcr-Abl in vitro. *Acta Pharmacologica Sinica*.

[18] Agrawal D. K., Mishra P. K. (2010). Curcumin and its analogues: potential anticancer agents. *Medicinal Research Reviews*.

[19] Lin L., Shi Q., Nyarko A. K. (2006). Antitumor agents. 250. Design and synthesis of new curcumin analogues as potential anti-prostate cancer agents. *Journal of Medicinal Chemistry*.

[20] Zhang Q., Zhong Y., Yan L.-N., Sun X., Gong T., Zhang Z.-R. (2011). Synthesis and preliminary evaluation of curcumin analogues as cytotoxic agents. *Bioorganic & Medicinal Chemistry Letters*.

[21] Gyuris M., Puskas L. G., Kanizsai I., Ozsvari B., Hackler L., Nagy L. I. (2013). Subtituted curcumin derivates, process for their preparation, and pharmaceutical compositions containing them. *HU*.

[22] Mitsiades N., Mitsiades C. S., Richardson P. G. (2003). The proteasome inhibitor PS-341 potentiates sensitivity of multiple myeloma cells to conventional chemotherapeutic agents: therapeutic applications. *Blood*.

[23] Karin M. (2006). Nuclear factor-*κ*B in cancer development and progression. *Nature*.

[24] Wright J. J. (2010). Combination therapy of bortezomib with novel targeted agents: an emerging treatment strategy. *Clinical Cancer Research*.

[25] Adams J., Kauffman M. (2004). Development of the proteasome inhibitor Velcade (Bortezomib). *Cancer Investigation*.

[26] Fabre C., Mimura N., Bobb K. (2012). Dual inhibition of canonical and noncanonical NF-*κ*B pathways demonstrates significant antitumor activities in multiple myeloma. *Clinical Cancer Research*.

[27] Hideshima T., Ikeda H., Chauhan D. (2009). Bortezomib induces canonical nuclear factor-*κ*B activation in multiple myeloma cells. *Blood*.

[28] Park J., Ayyappan V., Bae E.-K. (2008). Curcumin in combination with bortezomib synergistically induced apoptosis in human multiple myeloma U266 cells. *Molecular Oncology*.

[29] Zhang X.-Y., Bai Q.-X., Huang G.-S., Zhao H., Chen J.-J., Yang L.-J. (2011). Effect of curcumin in combination with bortezomib on proliferation and apoptosis of human multiple myeloma cell line H929 and its mechanism. *Zhongguo Shi Yan Xue Ye Xue Za Zhi*.

[30] Sung B., Kunnumakkara A. B., Sethi G. (2009). Curcumin circumvents chemoresistance in vitro and potentiates the effect of thalidomide and bortezomib against human multiple myeloma in nude mice model. *Molecular Cancer Therapeutics*.

[31] Mujtaba T., Kanwar J., Wan S. B., Chan T. H., Dou Q. P. (2012). Sensitizing human multiple myeloma cells to the proteasome inhibitor bortezomib by novel curcumin analogs. *International Journal of Molecular Medicine*.

[32] Palotas A., Puskas L. G., Kitajka K. (2004). The effect of citalopram on gene expression profile of Alzheimer lymphocytes. *Neurochemical Research*.

[33] Bai Q.-X., Zhang X.-Y. (2012). Curcumin enhances cytotoxic effects of bortezomib in human multiple myeloma H929 cells: potential roles of NF-*κ*B/JNK. *International Journal of Molecular Sciences*.

[34] Tzifi F., Economopoulou C., Gourgiotis D., Ardavanis A., Papageorgiou S., Scorilas A. (2012). The role of BCL2 family of apoptosis regulator proteins in acute and chronic leukemias. *Advances in Hematology*.

[35] Ramachandran C., Rodriguez S., Ramachandran R. (2005). Expression profiles of apoptotic genes induced by curcumin in human breast cancer and mammary epithelial cell lines. *Anticancer Research*.

[36] Pae H.-O., Jeong S.-O., Jeong G.-S. (2007). Curcumin induces pro-apoptotic endoplasmic reticulum stress in human leukemia HL-60 cells. *Biochemical and Biophysical Research Communications*.

[37] Gao M., Dong W., Hu M. (2010). GADD45*α* mediates arsenite-induced cell apoptotic effect in human hepatoma cells via JNKs/AP-1-dependent pathway. *Journal of Cellular Biochemistry*.

[38] Gottesman M. M., Pastan I. (1993). Biochemistry of multidrug resistance mediated by the multidrug transporter. *Annual Review of Biochemistry*.

[39] Sato H., Preisler H., Day R. (1990). MDR_1_ transcript levels as an indication of resistant disease in acute myelogenous leukaemia. *British Journal of Haematology*.

