# Supplementary material for: Curcumin and Its Analogue Induce Apoptosis in Leukemia Cells and Have Additive Effects with Bortezomib in Cellular and Xenograft Models
Source: Biomed Res Int. 2015 May 17;2015:968981. doi: 10.1155/2015/968981 (PMC4449904; doi:10.1155/2015/968981)
Supplement: Supplementary file 1 — "Supplementary Table: Normalized gene expression values (∆∆Ct) of all tested conditions. Curcumin and its analogue (C-150) induced expression changes in apoptotic and cell-cycle related genes as determined by high-throughput QPCR. Most of the affected genes showed similar changes suggesting that the related compounds act through similar signaling pathways. C-150 possessed dose dependent effects on certain apoptosis and cell cycle related genes in 40-fold lower concentration than curcumin which could be enhanced by coadministration with bortezomib. Red cells: at least 2 fold induction, green cells: at least 2 fold repression, nd: not detected transcript. [file 968981.f1.pdf]

Supplementary Table 1. Normalized gene expression values (ΔΔCt) of all tested conditions. Red cells: at least 2 fold induction, green cells: at least 2 fold repression, nd: not detected transcript.

	BTZ 20nM	Curc 25μM	Curc 25μM + BTZ 20nM	C-150 300nM	C-150 600nM	C-150 300nM+BTZ 20nM	C-150 600nM+BTZ 20nM
ABL 1	0.48	0.98	1.17	-0.32	0.55	-1.39	0.40
AKT1	-0.59	-0.43	-0.91	-0.59	0.03	-0.41	-0.72
ANAPC2	0.49	0.28	0.69	-0.78	-0.40	0.42	0.55
ANAPC4	-0.09	0.09	0.02	-0.26	-0.07	-0.39	-0.47
APAF1	0.10	0.93	0.73	0.12	0.39	0.30	0.17
ATM	1.01	0.12	0.51	-0.60	-0.21	0.60	0.30
ATR	-0.37	-0.61	-0.96	-0.12	-0.38	-0.79	-0.90
AVEN	-1.01	-0.12	0.65	-0.32	-0.27	-0.62	-0.41
BAD	0.42	1.01	1.59	-1.45	0.70	0.41	1.52
BAG1	-0.16	0.42	0.74	0.66	0.20	0.10	0.09
BAK1	-0.15	0.09	1.21	-1.75	-0.53	-0.23	0.74
BAX	0.89	0.54	0.16	0.78	0.29	0.45	-0.01
BAX	0.02	0.67	-0.01	0.33	0.65	0.56	-0.05
BBC3	-0.09	0.61	1.46	-1.57	-0.57	0.06	0.92
BCCIP	0.37	0.96	1.78	0.00	0.06	-0.15	0.01
BCL2	-1.57	-1.55	-2.83	-0.65	-0.38	-2.07	-2.60
BCL2	-1.73	-1.54	-2.47	-0.32	-0.17	-1.88	-2.39
BCL2L1	-0.83	0.43	0.30	-0.30	0.28	-0.08	-0.08
BCL2L10	0.01	0.79	2.05	-2.10	-0.71	0.05	1.21
BCL2L13	0.30	0.31	0.01	-0.05	0.39	0.50	0.35
BCL2L2	0.13	0.97	0.33	0.10	0.01	1.05	0.86
BID	0.01	0.22	0.01	0.31	0.24	0.02	-0.03
BIK	-1.11	0.40	0.84	-0.68	-0.44	-0.90	-0.57
BIRC2	0.66	1.15	0.67	0.08	0.54	0.86	0.41
BIRC3	-0.42	2.03	0.75	0.30	1.85	0.48	0.83
BIRC5	0.05	-0.08	-0.34	-0.63	-0.60	-0.58	-0.50
BIRC5	-0.55	0.13	-0.50	-0.21	-0.13	-0.27	0.10
BOK	nd	nd	nd	nd	nd	nd	nd
BRCA1	-0.06	-0.63	-1.21	-0.32	-0.22	-0.34	-0.22
BRCA2	-0.18	-0.44	-1.26	-0.71	-0.50	-0.31	-0.25
CAD	-1.69	-0.65	-1.94	0.28	0.14	-1.42	-1.64
CASP1	0.13	0.78	0.49	-0.46	-0.28	0.28	-0.36
CASP10	1.30	0.78	1.64	0.38	0.69	1.73	1.54
CASP12	-0.39	-1.35	1.11	-1.25	0.09	-0.08	0.86
CASP14	0.31	-1.12	0.90	-0.87	0.01	-0.21	-0.19
CASP2	-0.24	0.30	0.26	-0.64	-0.28	0.15	0.27
CASP3	0.26	1.03	1.20	0.46	0.72	0.68	0.99
CASP4	2.24	1.59	1.75	0.96	1.03	2.50	1.93
CASP5	nd	nd	nd	nd	nd	nd	nd
CASP6	0.41	0.70	0.99	0.45	0.38	0.62	0.45
CASP7	1.24	0.54	1.17	-0.27	-0.29	1.50	1.16
CASP8	1.23	0.31	0.12	0.42	0.63	1.42	1.16
CASP8P2	-0.31	0.21	-0.39	-0.17	0.13	0.25	0.09
CASP9	0.78	1.19	1.26	0.10	0.65	1.17	0.90
CCNA2	0.38	-0.04	-0.92	-0.36	-0.45	-0.14	-0.31
CCNA2	-0.74	-0.68	-0.35	0.11	0.04	-0.76	-0.82
CCNB1	0.05	-0.52	-0.99	-1.24	-0.50	-0.41	-0.25
CCNB2	0.33	0.13	0.33	-0.74	-0.40	-0.21	0.03
CCNC	1.10	0.47	1.05	-0.03	0.50	1.14	1.12
CCND1	-1.81	-1.37	-1.36	-1.13	-1.03	-1.85	-1.31
CCND2	-3.35	-3.71	-2.33	-1.09	-1.26	-4.55	-4.26
CCND3	0.88	0.54	0.17	-0.51	0.32	0.21	0.33
CCNE1	-0.16	-0.12	-0.60	-0.18	-0.26	-0.44	-0.73
CCNE2	0.85	0.86	0.55	0.04	-0.03	1.02	1.08
CCNF	-0.35	-0.18	-0.18	-0.68	-0.76	-0.64	-1.06
CCNG1	0.66	0.23	0.51	-0.18	0.15	0.33	0.39
CCNG2	1.11	1.22	1.18	-0.32	0.09	0.97	0.80
CCNH	0.23	0.06	0.09	-0.07	-0.32	-0.26	-0.14
CCNT1	0.50	0.56	1.53	-0.08	0.17	0.44	0.72
CCNT2	0.89	1.31	2.65	-0.29	0.09	0.75	1.17
CDC14A	-0.06	-0.16	0.78	-1.16	-0.75	-1.06	-0.51
CDC16	0.54	-0.06	0.36	-0.33	-0.26	0.47	0.39
CDC2	1.45	0.47	-0.05	0.12	0.18	0.64	0.26

CDC20	0.32	-0.24	-0.40	-0.51	-0.58	-0.17	-0.33
CDC25A	0.25	-0.23	0.54	-0.47	-0.59	-0.26	0.08
CDC25B	0.20	0.67	0.18	-0.64	0.12	-0.24	-0.07
CDC25C	0.58	0.14	-0.49	-0.43	-0.36	-0.02	-0.03
CDC34	0.71	0.42	1.01	0.04	-0.30	0.25	0.32
CDK2	0.30	0.21	0.75	-0.57	-0.40	-0.17	0.37
CDK4	0.10	-0.35	-0.64	-0.38	-0.23	-0.22	-0.44
CDK5R1	-0.02	0.91	1.68	-1.49	-0.82	-0.60	0.87
CDK6	-0.91	-0.74	-0.62	-0.83	-0.54	-0.90	-0.60
CDK7	0.70	0.49	1.10	-0.44	-0.45	0.26	0.55
CDK8	0.41	0.11	0.68	-0.35	-0.18	0.16	0.33
CDKN1A	3.25	3.23	4.02	-0.34	0.83	3.60	4.10
CDKN1B	0.54	0.41	0.05	-0.46	0.21	0.10	0.41
CDKN2A	-1.55	-0.29	-1.17	-1.82	-0.47	-0.47	-0.23
CDKN2B	nd	nd	nd	nd	nd	nd	nd
CDKN3	0.84	0.20	-0.52	0.01	-0.10	0.31	0.09
CFLAR	0.07	0.24	0.30	-0.04	0.86	0.15	0.12
CHEK1	0.54	-0.12	-0.39	-0.16	-0.40	0.08	0.05
CHEK2	0.34	-0.33	-0.42	-0.30	-0.51	-0.33	-0.60
CKS1B	0.26	0.17	0.55	-1.27	-0.94	-0.25	0.44
CKS2	0.63	-0.10	0.55	-0.67	-0.79	-0.32	-0.01
CRADD	-0.17	0.10	-0.60	-0.20	-0.24	-0.18	-0.44
CUL1	0.35	-0.25	0.81	-1.28	-0.37	-0.07	0.20
CUL2	0.25	0.42	0.56	-0.24	0.22	0.13	0.23
CUL3	0.11	-0.12	-0.11	-0.23	-0.15	-0.16	-0.28
DFFA	0.12	0.28	0.76	0.19	0.16	0.42	0.26
DIABLO	-0.26	0.48	0.51	0.25	-0.10	0.56	0.33
E2F1	0.09	0.37	0.35	-0.73	-0.33	0.10	0.21
E2F2	-0.10	0.86	-0.04	-0.33	-0.19	-0.01	0.20
E2F3	0.30	-0.09	0.93	-0.33	-0.15	0.40	0.39
E2F4	-0.37	-0.34	-0.22	-0.72	-0.23	-0.54	-0.41
E2F5	-0.33	-1.99	-2.16	-0.35	-0.67	-1.22	-1.49
E2F6	0.30	1.09	1.99	-1.11	-0.44	0.33	1.40
endoG	-0.98	-0.42	-0.70	0.17	-0.23	-1.60	-1.69
FADD	-0.02	0.71	1.34	-0.68	-0.20	0.32	0.75
FAM96A	-0.24	0.00	1.19	-1.16	-0.74	-0.13	0.48
FAM96B	1.22	1.08	1.30	0.88	0.78	1.28	1.00
FAS	0.40	0.25	0.37	0.33	0.16	0.58	-0.08
FASLG	-0.44	0.25	1.30	-2.23	-0.87	-0.34	0.55
GADD45A	2.15	1.39	2.79	-1.04	-0.79	2.11	3.23
GTF2H1	-0.20	0.29	0.44	-0.17	-0.17	-0.15	0.28
GTSE1	0.45	0.13	-1.12	-0.47	-0.28	0.17	-0.05
HMGB1	-1.18	-0.99	-0.60	-0.06	-0.53	-0.80	-1.22
HRK	-0.86	0.57	1.65	-1.67	-0.74	-0.34	0.83
HSP90B1	0.65	-0.48	0.16	-0.13	-0.37	0.74	0.21
HTRA2	-0.34	-0.33	-0.03	-0.43	-0.08	-0.18	0.07
JUN	4.31	2.65	4.71	-0.49	-0.11	4.16	4.29
KNTC1	0.66	-0.44	-0.90	-0.11	-0.10	0.41	0.32
KPNA2	-0.01	0.09	-0.03	-1.18	-0.81	-0.24	-0.61
LGALS3	0.99	1.70	1.64	0.17	0.57	1.26	1.24
LRDD	-0.16	0.01	-0.39	0.86	0.46	-0.23	-0.45
MAD2L1	0.51	-0.14	-0.64	-0.05	-0.23	0.05	0.07
MAD2L2	0.24	-0.12	-0.28	-0.18	-0.36	-0.40	-0.25
MCL1	0.43	0.86	1.79	-0.11	-0.04	0.97	0.76
MCM2	-0.52	-0.74	-1.25	-0.58	-1.27	-0.83	-0.85
MCM3	-0.90	-0.39	-0.66	-0.59	-0.81	-0.86	-0.98
MYC	-2.25	-4.09	-3.13	-0.52	-0.69	-3.05	-3.04
NFKB1	-0.73	0.22	-0.10	-0.22	0.35	0.05	-0.05
NFKB2	1.55	1.91	1.96	0.86	1.23	2.12	1.78
NGFR	nd	nd	nd	nd	nd	nd	nd
PCNA	0.41	-0.03	-0.67	0.36	-0.36	-0.18	-0.35
PLK1	0.08	0.08	-0.49	-0.43	-0.39	-0.43	-0.43
PMAIPI	1.97	0.87	1.99	-3.38	-0.72	0.53	2.85
PTEN	0.40	0.47	0.93	0.16	0.75	0.70	0.50
RAD1	-0.13	-0.05	1.53	-1.55	-0.56	0.09	0.76
RAD17	0.28	0.31	0.56	-0.69	-0.28	-0.15	0.29
RAD51	0.38	0.27	-0.21	0.08	-0.32	0.12	-0.08
RAD9A	0.84	0.86	1.34	-0.05	0.31	0.90	1.07
RB1	1.27	0.83	0.31	0.50	0.49	0.83	0.35
RBL1	0.03	0.03	0.72	-0.18	-0.45	-0.08	-0.41

RBL2	0.24	-0.05	-0.14	-0.18	-0.08	-0.05	-0.24
REL	-0.32	0.16	1.35	-1.72	-0.41	-0.17	0.82
RELA	0.49	0.77	0.98	0.15	0.36	0.96	0.86
RELB	0.56	1.52	1.21	-0.04	0.79	1.34	1.21
SKP2	-1.03	-1.16	-1.47	-0.58	-0.52	-1.44	-1.45
SOCS2	-0.12	0.47	1.54	-2.05	-0.39	-0.06	0.80
SOCS3	nd	nd	nd	nd	nd	nd	nd
STAT1	0.58	0.56	0.37	0.46	0.57	0.87	0.34
STAT5A	-1.60	-0.28	-0.75	0.28	-0.11	-1.43	-1.81
STAT5B	-0.22	0.08	0.42	-0.07	0.22	-0.08	-0.29
TFDP1	-0.93	-0.99	-1.48	-0.51	-0.65	-1.19	-1.24
TFDP2	1.17	0.06	0.70	-0.38	-0.48	0.90	0.78
TNF	-0.78	0.93	1.37	-1.37	-0.56	0.51	1.34
TNFRSF10A	0.95	1.49	1.39	0.23	0.52	1.27	1.59
TNFRSF10B	1.06	1.34	2.13	0.42	0.42	1.73	2.62
TNFRSF10C	-0.43	2.03	0.82	-0.83	-1.06	-0.61	-1.06
TNFRSF10D	0.30	0.93	1.83	-0.22	0.17	0.64	1.07
TNFRSF1A	-0.49	-0.15	0.36	-0.60	-0.12	-0.11	-0.10
TNFRSF1B	-0.43	0.42	-0.10	0.40	0.47	-0.54	-0.63
TNFRSF21	0.14	0.35	-0.14	0.35	0.82	0.50	0.18
TNFRSF25	-0.03	0.76	1.57	0.76	0.30	0.08	1.51
TNFSF10	-1.58	0.97	-0.79	-0.05	0.83	-0.47	-0.68
TNFSF11	nd	nd	nd	nd	nd	nd	nd
TNFSF8	nd	nd	nd	nd	nd	nd	nd
TP53	-0.07	-0.23	0.61	-1.30	-0.82	-0.31	0.24
TP53	-0.37	-0.07	0.76	-0.72	-0.51	-0.44	0.28
TP53I3	nd	nd	nd	nd	nd	nd	nd
TRAF1	2.23	2.57	2.70	-0.29	1.12	3.10	3.40
TRAF2	0.10	0.45	0.55	-0.19	-0.06	0.28	0.05
TRAF3	0.29	0.21	0.60	-0.21	-0.24	0.25	0.07
TRAF5	-0.62	-0.31	-0.18	-0.43	-0.10	-0.02	-0.14
TRAF6	0.75	1.65	2.18	-0.14	0.30	1.33	1.27
TRAF7	-0.38	0.07	0.33	0.17	-0.07	-0.04	-0.50
WEE1	0.34	-0.20	-0.63	-0.37	-0.42	-0.24	-0.08
